# Exploring patient perspectives of barriers and facilitators to participating in hospital-based pulmonary rehabilitation in patients diagnosed with non-small-cell lung cancer treated with curative intent

**DOI:** 10.1007/s11845-024-03855-7

**Published:** 2025-01-17

**Authors:** Denise Doyle, Matthew O’Brien, Rosemary Murphy, Aidan O’Brien, Deirdre McGrath, Dervla Kelly

**Affiliations:** 1 Faculty of Education and Health Services, School of Medicine, University of Limerick, GarraunLimerick, V94 T9PX Castletroy, Co Ireland; 2 University Hospital Limerick, Limerick, Ireland

**Keywords:** Barrier, Facilitator, Lung cancer, Participation, Pulmonary rehabilitation

## Abstract

**Background:**

Pulmonary rehabilitation (PR) is increasingly offered to patients who have undergone lung resection for non-small-cell lung carcinoma (NSCLC) as it can improve exercise tolerance and quality of life. However, designing and implementing such a complex multidisciplinary programme has its challenges.

**Objective:**

This study aims to explore perspectives of patients offered PR services post-lung resection for NSCLC to gain an understanding of the potential barriers and facilitators behind implementing and designing PR programmes.

**Methods:**

The PR programmes were offered in an outpatient rehabilitation gym at University Hospital Limerick, Ireland. Patients attending routine follow-up appointments post-lung cancer resection during the period of the study were identified and based on inclusion criteria were invited to participate in semi-structured interviews (focus groups). Interviews with patients who participated in the PR programme (*n* = 8) and those who declined (*n* = 4) were transcribed and major themes identified using normalisation process theory (NPT analysis) before interpretation of the themes in context.

**Results:**

Across 4 focus groups that were conducted, the important barriers identified were the presence of comorbidities, location, perceived lack of benefit, and a fear of causing harm. Relevant facilitators included the prospect of acquiring new knowledge, optional access to psychological support, peer interaction/social support, individualised, tailored programmes, and a holistic approach to recovery as well as goal setting and accomplishment.

**Conclusions:**

Patient participation in PR has been shown to be influenced by both external and intrapersonal variables. The barriers and facilitators experienced by the patients in this study contribute to the existing knowledge of the patient experience of pulmonary rehabilitation and can help to inform clinical practice and future research.

## Introduction

Lung cancer is the third most common cancer diagnosed in Ireland with non-small-cell lung cancer (NSCLC) being the most common histopathological subtype (National Cancer Registry, 2022). Surgical resection remains the gold standard treatment in early NSCLC [1]; however, the surgery itself can cause significant pulmonary function impairment [2].

Pulmonary rehabilitation (PR) plays an important part in recovery from treatment post-lung resection surgery. PR is a comprehensive programme which amalgamates exercise training with psychological and behavioural tools to rebuild the functional capacity of patients [3]. Several studies have demonstrated the safety and efficacy of PR whilst documenting improvements in exercise capacity, muscle strength, dyspnoea, fatigue, and health-related quality of life post-lung resection surgery [4–7]. Despite a wealth of evidence demonstrating the effectiveness of PR post-lung resection surgery, implementation in clinical practice remains suboptimal. Furthermore, programme implementation in small, local cancer centres has been under researched to date. Research highlights that the successful implementation of evidence into practice requires tailoring of rehabilitation programmes to a specific setting with local stakeholders so that barriers to implementation in the local context can be understood and addressed [8, 9].

While previous research has highlighted some barriers to implementation of lung cancer rehabilitation interventions [4, 10, 11] the main challenge in this area today is to get stakeholder input and design and tailor strategies that address these barriers. Taking a participatory health research approach to implementation problems has the potential to improve the uptake, adherence, and sustainability of healthcare interventions [8, 9].

Participatory health research strives to maximise the participation of end-users in all stages of the research process. Research is not done “on” people as passive subjects providing “data” but “with” them to provide relevant information for improving their lives [12]. Applying these methodologies to pulmonary rehabilitation in cancer could provide deeper understanding of what makes these programmes work.

Despite a favourable prognosis and extensive evidence suggesting the potential positive effects of PR, patients with NSCLC still suffer from unmet rehabilitation needs. Designing and implementing a successful PR programme on a local level is logistically challenging and involves an extensive multidisciplinary team. This study aims to explore the potential barriers and facilitators to the implementation of such programmes and to identify strategies to successful operationalisation of PR programmes for those who have undergone curative resection of NSCLC.

## Methods

### Study design

A qualitative study using semi-structured focus groups with patients who had undergone resection for NSCLC was performed. A total of 12 patients were interviewed for this study, all of whom were offered PR with no obligation to partake. Eight of the study participants opted to partake in the PR programme offered in a physiotherapy gym located on the grounds of an acute hospital.

Throughout this research endeavour, the COnsolidated criteria for REporting Qualitative research (COREQ) were used to perform and report this study [13]. Furthermore, we have employed normalisation process theory (NPT) as a framework to assess the descriptions of exercise behaviours and healthcare practices from the people recovering from lung cancer and health professionals in order to identify facilitators and barriers to rehabilitation exercise, the environment (hospital), and if possible, any strategies that may help with implementation. Developed by May and Finch, NPT is a framework that can be applied across healthcare contexts and topics to explain, and potentially shape, implementation processes [14]. NPT has four components that provide a framework for this process. By incorporating theory, we hope this can help us overcome barriers and enhance facilitators and evaluate the implementation in the future.

### Participants

Having gained ethical approval, patients with a diagnosis of NSCLC who had undergone lung resection were identified. Patients attending routine follow-up appointments during the period of the study (September–December 2022) were invited to participate by a administrative gatekeeper not directly involved in their care. The study inclusion criteria was those aged 18 years or over with a diagnosis of lung cancer who were treated with surgery between the period of 1st January 2019 and 31st December 2020. Twelve participants volunteered to take part in 3 focus groups. Recruitment was stopped after the third focus group as there were no new themes being identified in the interviews. No repeat interviews were performed. Of the 12 focus group participants, 8 opted to partake in the PR programme. Participation was voluntary and no disadvantages arose from non-participation. Participants were supplied with information on the objectives of the focus group interviews, and written informed consent was obtained from all participants. Background information on the interviewees was collected regarding age, gender, comorbid conditions, and smoking status was also collected (Table 1).

**Table 1:** Patient demographics

		Focus group 1 (*N* = 2)	Focus group 2 (*N* = 6)	Focus group 3 (*N* = 1)	Focus group 4 (*N* = 3)
Date of focus group		19.11.2022	24.11.2022	28.22.2022	15.12.2022
Gender	M	1	4	0	0
	F	1	2	1	3
Age	Mean (SD)	64.5 (2.12)	65.2 (15.5)	83	68.6 (8.4)
Smoking history	Current smoker	0	0	0	0
	Ex-smoker	2	6	1	1
	Never smoked	0	0	0	2
COPD status	Yes	2	3	0	1
	No	0	0	0	0

### Setting

The gym is an outpatient rehabilitation gym located on the site of University Hospital Limerick, Ireland. The physiotherapists provided goal-directed pulmonary rehabilitation to individuals who had undergone surgery for NSCLC. In line with infection control policy, groups of 6 individuals were scheduled for rehabilitation sessions to enable adequate social distancing and all participants were required to wear masks. All participants were screened prior to and after each session for wellness, suitability to partake in exercise and BP, SpO_2_, and HR were recorded pre- and post-sessions. Participants were also provided with additional education sessions along with the option to avail of counselling. From here, individuals are discharged home to continue with exercise as they see fit. An image of the rehabilitation gym and a list of equipment available is provided in Appendix A.

### Research team

The multidisciplinary research team consisted of 6 researchers. Two consultant physicians, one pharmacist, one medical student, one physiotherapist, and one advanced nurse practitioner. Relationships were not established between the participants and researchers prior to the study. Brief introductions were had prior to the commencement of the focus groups to provide some information about the study and its potential usefulness.

### Data collection

Focus groups were conducted by 4 members of the research team. Focus groups were conducted between November and December 2022 with a total sample size of *N* = 12. Three of the focus groups were conducted in person and one via zoom. The interviews lasted approximately one hour. A critical review of available literature on PR after lung cancer surgery was conducted to gain an adequate understanding of the subject, and from this, an appropriate list of questions was compiled to ask participants during each interview. All of the focus groups were digitally recorded and transcribed verbatim by a member of the research team. The transcripts were pseudonymized and given a consecutive ID number from 1 to 12. Transcripts were not returned to participants for comment and/or correction.

### Data analysis

Four anonymised transcripts (1 for each focus group) were reviewed and independently coded by the first and last author using an open coding procedure. A coding frame for our NPT deductive analysis was prepared separately and data related to each construct was entered onto a data coding sheet in excel (available upon request).

## Results

### Participant characteristics

Focus group interviews were conducted with 12 participants in total (7 women and 5 men; Table 1). The first and last authors were present in all interviews and participated in conversation to varying extents; however, the findings reported here focus on patient perspectives and therefore only include quotes from the patients themselves. The median age of the participants was 69 years. Ten of the participants are now ex-smokers and 2 of the participants lifelong non-smokers. Notably, 6 of the participants have been diagnosed with COPD.

### Themes

Four themes emerged from this analysis: understanding of the programme, taking part, making it happen, and possible modifications to improve implementation.

#### Theme 1 (coherence): understanding of the programme

The phrase ‘pulmonary rehabilitation’ had been unfamiliar to the participants prior to attending the rehabilitation programme; despite the fact, it was going to be a key aspect of their recovery:

‘I didn’t realise what I was going to be doing as such. I knew it was an exercise programme but it was really as the weeks went on we got some more information. If you knew how difficult it would have been by the end then maybe you wouldn’t have come in!’ (Participant 11, focus group 4).

A lack of awareness about the programme paired with varying attitudes towards rehabilitation lead to some confusion as to whether the programme was warranted or not:

**‘**I really didn’t think I needed it’ (Participant 9, 3rd focus group).

Some participants shared their initial apprehension regarding feeling short of breath and some expressed the feeling that they would not have been able to partake in the PR programme for several months post resection:

‘I was nervous because I was questioning whether I would do harm to myself or if I was going to overdo it’ (Participant 10, focus group 4).

It was very important to the participants that the importance and benefits of pulmonary rehabilitation was communicated to the participants throughout the programme:

‘They would have told us that exercise is huge and it’s such an important thing in general but that it’s particularly good for people who have had cancer and for the prevention of cancer and recurrence which really helps you want to do it a bit more.’ (Participant 11, focus group 4).

#### Theme 2 (cognitive participation): taking part

Participants valued an approach from staff that supported them to discover their own limitations within a safe environment and, in so doing, increased their self-confidence. It was noted that the exercise programme were individualised and tailored as needed:

‘I was going through a bad period of back and hip pain but the physiotherapist was able to amend my exercises so I can still get the benefit from them without excess pain’ (Participant 1, focus group 1).

‘It made me realise then that I wasn’t doing enough at home and it’s okay to push yourself, that a little bit more, and If you don’t feel a little bit breathless, you’re really not doing much work. I know now I can push myself a bit more’ (Participant 11, focus group 4).

A high level of peer support was also highlighted as a main benefit of the programme within all groups:

‘I met so many people who had survived cancer with such positive attitudes and I found it really encouraging’ (Participant 3, focus group 2).

‘The social aspect is huge, and we all have something in common and we can speak to each other 1 to 1’ (Participant 2, focus group 1).

‘Oftentimes, sickness isn’t discussed which provides the mental reprieve that individuals living with cancer need.’ (Participant 2, focus group 1).

#### Theme 3 (collective action): making it happen

Designing and implementing a PR programme involves a wide multidisciplinary team including doctors, nurses, physiotherapists, and dieticians. These valued members of staff were available to support the programme:

‘The exercise sessions were then followed by short information sessions about nutrition, exercise, mindfulness, mental health, and respiratory disease management on alternate weeks, all of which I really enjoyed’ (Participant 1, focus group 1).

The option to have psychological support was also available:

‘I came in here to a councillor about 6 weeks after my op and they were absolutely amazing. The nurse practitioner made me aware of this service (Participant 2, focus group 1).

However, it was noted that some patients lacked the ability to partake in this programme due to other comorbid conditions:

‘I actually couldn’t do the walking as the veins in my leg are very clogged and I wasn’t a candidate for stents. What I do now is go to the pool three times a week for 40 min and I walk constantly for the 40 min. But there was a time when I couldn’t get around the supermarket.’ (Participant 5, 2nd focus group).

Some patients who were offered to join the PR programme were unable to due to the location of the hospital along with conflicting responsibilities:

‘I was living in west Clare and I’d say if I had been in Dublin, I could have gone to any classes offered to me, but of course it wasn’t practical.’ (Participant 9, 3rd focus group).

‘I wouldn’t have had the time between looking after my mother and everything else’ (Participant 4, focus group 2).

#### Theme 4 (reflexive monitoring): possible modifications to improve implementation

Participants agreed that partaking in the programme was valuable. The content of the talks was found to be helpful, particularly information relating to the use of inhalers and diet:

‘I found learning about inhaler technique particularly useful. There is a whole process around taking inhaled medication correctly I was unaware of. I was also educated about the use of a spacer and this helps me get the correct dose of medication. Also, it was recommended that rinsing out my mouth after taking the medication would prevent oral thrush.’ (Participant 1, focus group 1).

‘I thought the dietician was great. I’d be hearing this and that about certain foods and she explained to me there was no scientific evidence behind a lot of what I had heard but she explained the importance of fruit and veg and I have been trying to get a lot more of it in.’ (Participant 11, focus group 4).

Furthermore, patients discovered their own true limitations:

‘It made me realise then that I wasn’t doing enough at home and it’s okay to push yourself, that a little bit more, and If you don’t feel a little bit breathless, you’re really not doing much work. I know now I can push myself a bit more’ (Participant 11, focus group 4).

## Discussion

The main results of this qualitative study highlighted the need to continue with relevant facilitators which included optional access to psychological support, social support, tailored programmes, and educational sessions to allow for acquisition of new knowledge. The data also highlighted that important barriers to the effective implementation of a PR programme were patient comorbidities, location, perceived lack of benefit of PR, and a fear of causing harm. The findings of this study will be discussed in relation to the existing literature.

A significant concern noted across the literature and in this study is a lack of awareness about PR programmes and their potential benefits [11, 15]. In 2014, Rochester and colleagues developed a survey to better understand patients perspectives on PR. Of 1685 respondents, 40% reported that their healthcare provider never told them about PR programmes [16]. It has already been suggested in the literature that patient advocacy groups develop education materials for people living with chronic respiratory disease regarding the process and benefits of PR [10]. Furthermore, the existing literature alongside this analysis confirms the need for greater healthcare professionals’ knowledge and awareness of PR to foster patient referrals.

Location of PR programmes and access difficulties are highlighted as barriers to attendance at PR programmes in this study and across the literature [15, 17]. Four of twelve individuals in this analysis chose not to attend the PR programme due to conflicting responsibilities, comorbid conditions that would hamper their ability to partake, access difficulties, and feeling that the course was ‘not needed.’ Of the 8 individuals interviewed who chose to partake, there was a 100% completion rate. Travel to a PR centre along with parking charges may act as a barrier to attendance or completion of a programme. In future, a larger number of PR centres would certainly act as a facilitator to future uptake.

There is a lack of consensus across the literature about the best time to enrol in a rehabilitation programme post-treatment and no specific guidelines have been set. Rather, it has been suggested that whilst the timing of rehabilitation is crucial, it must be tailored to the individual patient and their disease process [18]. Many of the participants in this study echoed the fact they felt they would have been unable for as intense a programme until many months after the surgical resection, with several of these patients partaking in the PR programme 6–12 months post-surgery. Some patients may find it distasteful to discuss rehabilitation prior to surgery when facing a life-threatening illness. On the other hand, emphasising a proactive approach at an early time is fundamental to recovery [10].

The diagnosis and treatment of lung cancer negatively affect patients in terms of the physical and emotional well-being [2]. Therefore, when these patients are presented with a unique opportunity to improve their health status, they must have trust in the programmes ability to ‘do no harm.’ Not only has the safety and efficacy of PR been widely researched and supported [6, 19] the strong social aspect of the course can improve mental health and establish social support networks. Those who have participated in PR report enjoying the social aspect of training together, ultimately serving to encourage and motivate one another [20]. Besides the individuals with lung cancer or chronic respiratory diseases, the caregivers might also find the desired social support in the PR setting [20].

### Strengths and limitations

Our study has strengths and limitations. The use of implementation science allowed for evaluation of evidence which reflected community and stakeholder priorities. Nonetheless, this is a single-centre study in a large teaching hospital. The aim of this paper was to explore patient perspectives. However, to get a full understanding of perceived barriers/facilitators to PR implementation, the thought and opinions of the wider multidisciplinary team should be sought, some of whom contributed to this paper.

### Further research suggestions

Prospective studies assessing the psychological and physical outcomes of PR involving large sample size comprising patients with different cancer stages and comparing PR programmes would also be hugely informative. Furthermore, as the role of prehabilitation is a growing area in cancer care, it would be beneficial if the impact of prehabilitation on engagement with, and benefit from, post-resection PR programmes and on patient quality of life and survival was defined.

## Conclusion

In this analysis, we identified that the most striking barriers to participation in PR programmes are the presence of comorbidities, location, perceived lack of benefit, and a fear of causing harm. As a result, strategies aimed at facilitating better patient education about the importance of PR alongside education of physicians on the importance of referrals to PR programmes would improve uptake rates and positive outcomes for patients and their families.

Regardless, recovery after resection of NSCLC remains a major challenge. This challenge is only exacerbated by the growing burden of obesity, diabetes, COPD, and other chronic diseases. Pulmonary rehabilitation programmes implemented by multidisciplinary teams can offer these patients a unique opportunity to improve their health status and quality of life. As we are now entering the era of personalised medicine, future large-scale studies will almost certainly be required to identify specific rehabilitation programmes most suited for specific individuals and their state of health.

## Data Availability

The data that support the findings of this study are available from the corresponding author, [DD], upon reasonable request.

## Abbreviations

NSCLC: Non-small-cell lung cancer
PR: Pulmonary rehabilitation
